# Genetic diversity in three invasive clonal aquatic species in New Zealand

**DOI:** 10.1186/1471-2156-11-52

**Published:** 2010-06-18

**Authors:** Carla Lambertini, Tenna Riis, Birgit Olesen, John S Clayton, Brian K Sorrell, Hans Brix

**Affiliations:** 1 Department of Biological Sciences, Plant Biology, Aarhus University, DK-8000 Århus C, Denmark; 2 National Institute of Water and Atmospheric Research, P.O. Box 11115, Hamilton, New Zealand

## Abstract

**Background:**

*Elodea canadensis, Egeria densa *and *Lagarosiphon major *are dioecious clonal species which are invasive in New Zealand and other regions. Unlike many other invasive species, the genetic variation in New Zealand is very limited. Clonal reproduction is often considered an evolutionary dead end, even though a certain amount of genetic divergence may arise due to somatic mutations. The successful growth and establishment of invasive clonal species may be explained not by adaptability but by pre-existing ecological traits that prove advantageous in the new environment. We studied the genetic diversity and population structure in the North Island of New Zealand using AFLPs and related the findings to the number of introductions and the evolution that has occurred in the introduced area.

**Results:**

Low levels of genetic diversity were found in all three species and appeared to be due to highly homogeneous founding gene pools. *Elodea canadensis *was introduced in 1868, and its populations showed more genetic structure than those of the more recently introduced of *E. densa *(1946) and *L. major *(1950). *Elodea canadensis *and *L. major*, however, had similar phylogeographic patterns, in spite of the difference in time since introduction.

**Conclusions:**

The presence of a certain level of geographically correlated genetic structure in the absence of sexual reproduction, and in spite of random human dispersal of vegetative propagules, can be reasonably attributed to post-dispersal somatic mutations. Direct evidence of such evolutionary events is, however, still insufficient.

## Background

Invasive plants are of evolutionary interest because they are successful in areas where they have been introduced, sometimes in spite of limited genetic variation compared to their native range. One reason for this success may be multiple introductions, representing genetically distant genotypes. Further, an introduced species can overcome the negative effects of genetic bottlenecks and create genetic variation in the introduced range when genotypes from different geographical areas are brought together, giving rise to genetic combinations resulting in novel genotypes not present in the native range. In the case of *Phalaris arundinacea *L. in North America, the genetic variation is even comparable to that found in the native European range [1]. Apparently, however, many plant species become successful invaders in spite of very limited or non-existing genetic variation. This is the case in perennial clonal species, which establish themselves and disperse by vegetative propagation. Examples are the tropical American aquatic species *Alternanthera philoxeroides *(C. Martius) Griseb. [2] and *Eichornia crassipes *(C. Martius) Solms-Laub. [3] in their introduced range in China.

Clonal growth is often considered an evolutionary dead end, as defined by Stebbins [4] for self-fertilized plants. Somatic mutations do occur, although not frequently, and their role in the evolution of clonal species has been extensively modelled [5].According to the somatic mutation theory of clonality of Klekowski [6,7] plants accumulate mutations in meristematic cells with age. Such mutations reduce the likelihood of sexual reproduction and after a certain time, evolutionary changes depend exclusively on somatic mutations with advantageous phenotypes. It is assumed that sexual reproduction reduces genetic diversity by preventing somatic mutations from being transmitted to the offspring [8]. In this respect the structure and organization of meristems (monopodial vs. sympodial branching) affects the somatic mutation load that can be transferred to gametes [8]. The concept of population as a group of interbreeding individuals is not obviously applicable to clonal species. A "population" of a clonal species could be said to be represented by a group of stands of which each may consist of one or more genotypes (clones). In contrast to the situation in obligately outbreeding sexually reproducing organisms, monoclonal "populations" are common [9] and even in the case of clonal diversity, Hardy-Weinberg equilibrium cannot be expected to occur, as sexual reproduction may occur sporadically, if at all.

The North Island of New Zealand (NZ) is a suitable area for the study of genetic variation patterns of invasive species because, being an island, it is naturally delimited, and the site of many plant invasions that have been thoroughly monitored and documented. In addition, NZ has only recently been colonized by Europeans and therefore provides a unique opportunity to study recent invasion pathways and spread, as well as habitat adaptation. *Elodea canadensis *Michaux, *Lagarosiphon major *(Ridley) Moss and *Egeria densa *Planchon are invasive in NZ and in many other parts of the world. They are all dioecious Hydrocharitaceae and, like many submerged clonal macrophytes, rarely achieve sexual reproduction even in the native range [10]. *Elodea canadensis *is native to North America and was introduced in NZ in 1868 [11]. *Egeria densa *is native to South America (Brazil, Argentina and Paraguay) and was introduced in NZ in 1946 [12]. *Lagarosiphon major *is native to South Africa and was first recorded in NZ in 1950 [12]. Only female plants of *E. canadensis *[13] and *L. major *[14] and male plants of *E. densa *[14] have been found in NZ. One single record from 1988 (I.M. Johnstone, NZ Electricity Department, pers. comm.) also reported male plants of *E. canadensis *from the Waingaro River (Waikato Region, Central North Island) but this appears to be an isolated record, as male plants have not been featured in subsequent surveys of the aquatic vegetation throughout NZ. Even though both *E. canadensis *sexes might potentially co-exist in some waters, seeds have never been observed in any of the three species [14]. This limits interbreeding opportunities and excludes the possibility of hybridization between the three not so distantly related species, as well as agamospermy. The similar histories, the shared geographical setting and the apparent absence of sexual reproduction make this system suitable for the study of differentiation in invasive clonal species. The distribution and history of spread of these species in the North Island, documented by historical floristic records of first introduction date, suggest one single introduction for both *E. densa *and *L. major *[12], but this has never been critically tested with molecular markers. The same is true also for *E. canadensis *[11,13], but in this case the floristic records are less complete, and historical scenario for the history of the species in NZ is even more speculative. The possibility of multiple introductions of *E. canadensis *was pointed out by Thomson [15] who provided evidence for repeated introduction of live fresh-water fish to New Zealand from Tasmania, where *E. canadensis *was already established at the time. In this study we attempt to determine the number of introductions and evaluate the role of somatic mutations and dispersal as possible sources of the genetic variation patterns observed. We have used AFLPs (Amplified Fragment Length Polymorphism; [16]), because of the high number of DNA markers that this technique is able to detect. This aspect is important when identifying identical genotypes, and when assessing small genetic differences between clones. We employed some precautions to recognize and exclude possible AFLP artefactual polymorphic fragments.

## Results

**Figure 1:**
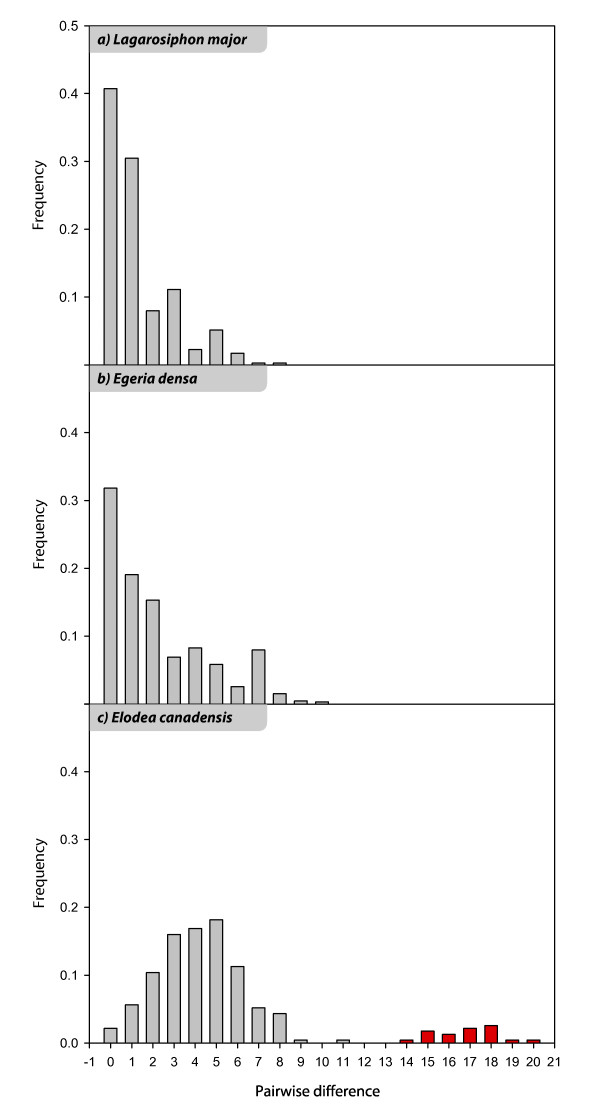
**Spectra of pairwise genetic differences between NZ genotypes of 1a) *Lagarosiphon major*; 1b) *Egeria densa*; 1c) *Elodea canadesis***. In *E. canadensis *pairwise differences from 0 to 11 are between NZ genotypes (gray), from 14 to 20 are between the NZ genotypes and an *E. canadensis *clone from Denmark (red).

Similar pairwise genetic difference ranges were found in the three species (0- 11 in *E. canadensis*, 0-10 in *E. densa *and 0-8 in *L. major*), whereas their frequencies showed different patterns (Figure 1). In *E. densa *and in *L. major *most of the samples belonged to the same genotype (0 pairwise differences) and most of the different samples had one pairwise difference. In *E. canadensis *a few samples were genetically identical and the spectrum of pairwise difference frequencies showed a normal distribution with highest frequency of samples differing from each other in 5 DNA fragments. Pairwise genetic differences with a genotype of *E. canadensis *from Denmark ranged between 14 and 20 DNA fragments, indicating an higher extent of genetic differentiation between the European genotype and all the NZ samples, and a net separation of their pairwise difference frequencies in the spectrum.

**Figure 2:**
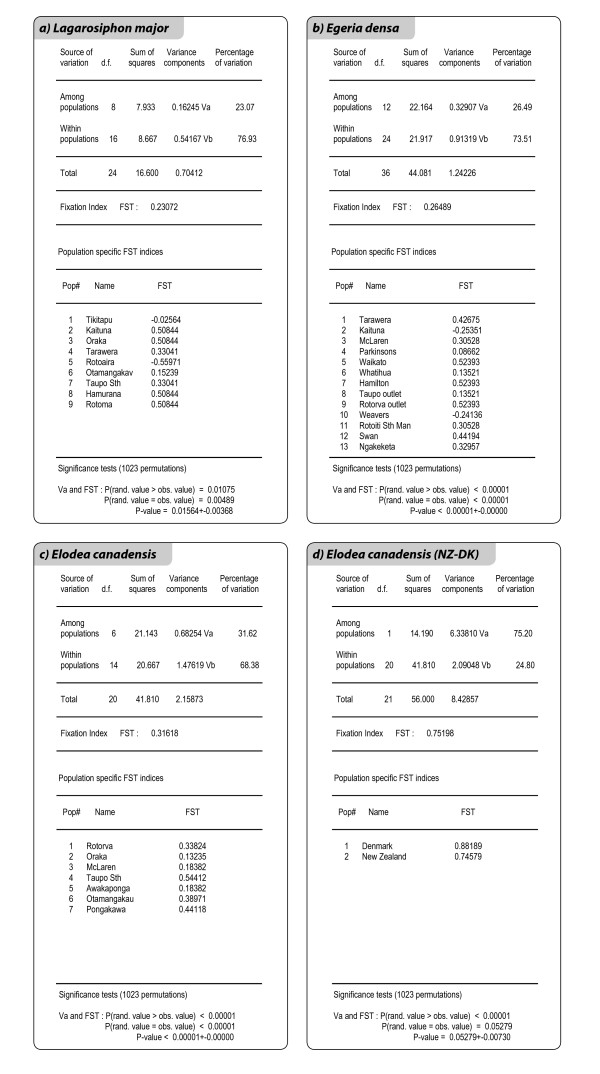
**AMOVA within and among NZ populations of 2a) *L. major*, 2b) *E. densa*, 2c) *E. canadensis*. 2d) AMOVA within and among NZ samples, considered as one single population, and one Danish genotype of *E. canadensis***. Population specific FST indices indicate how much each population contributes and deviates from the weighted average FST.

The analysis of molecular variance showed higher percentages of genetic variation within populations than among populations in all species. Similar levels of population differentiation were found in *E. densa *(average Fst = 0.27; p-value = < 0.001) and in *L. major *(average Fst = 0.23; p-value = 0.02), whereas *E. canadensis *populations showed higher genetic structure (average Fst = 0.32; p-value < 0.001). The population specific Fst indices (Figure 2) show how much the Fst of each population contributes and deviates from the weighted average Fst. Monoclonal populations showed the highest specific Fst, but these values are affected by the clonal nature of the populations. Heterozygosity, calculated as covariance component of the total variance, is "zero" in monoclonal populations and Fst corresponds to the total heterozygosity of the sample set, or is very close to it, depending on the algorithm used. Even though Fst values cannot be interpreted in terms of gene flow among populations, the different average Fst values in the three species give an idea of differences in genetic structure at the population level. The population comparison test and the exact test of population differentiation showed no population differentiation in *E. canadensis*. In *L. major *the Tikitapu and Otamangakau populations had a significant pairwise Fst of 0.35 (p-value = 0.03). In *E. densa *the McLaren and Swan populations had a significant pairwise Fst of 0.82 (p-value = 0.04 ). Population differentiation was not confirmed by the exact test of population differentiation in both species. Genetic differentiation between the NZ *E. canadensis *populations and the Danish genotype was 0.75 (p-value = 0.05), indicating that the NZ genotypes were more similar to each other than they were to the Danish clone. Nei's unbiased minimum genetic distances ranged between 0 and 0.01 between all pairs of populations in *L. major*. In *E. densa *they ranged between 0 and 0.06; however, only one population (McLaren) had genetic distances over 0.02 with all the other populations. By excluding McLaren, the range decreased to 0 - 0.02. Pairwise genetic distances among *E. canadensis *populations ranged between 0.01 and 0.08. Nei'unbiased minimum genetic distance was 0.17 between the NZ *E. canadensis *clones and the Danish genotype. The genetic distance values appeared to better reflect the genetic similarities among the populations than the Fst values and confirmed differences in genetic structure in the populations of the three species.

**Figure 3:**
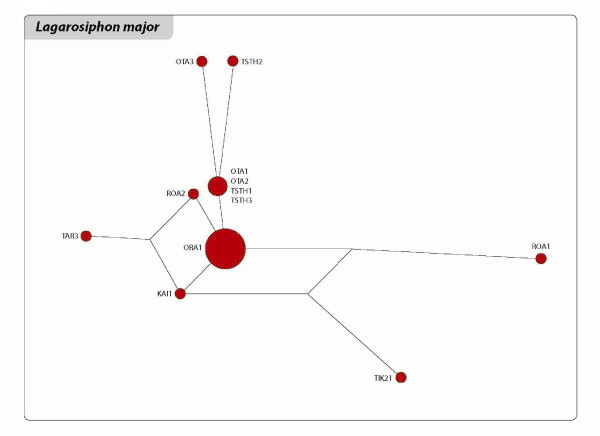
**Reduced median network of *Lagarosiphon major***. The size of terminal nodes is proportional to genotype frequencies. Samples are labeled with population abbreviation (see Abbreviations) and sample number (1-3). The genotypes represented by the big terminal node "ORA1" are the same as those indicated in the small frame of Figure 4.

**Figure 4:**
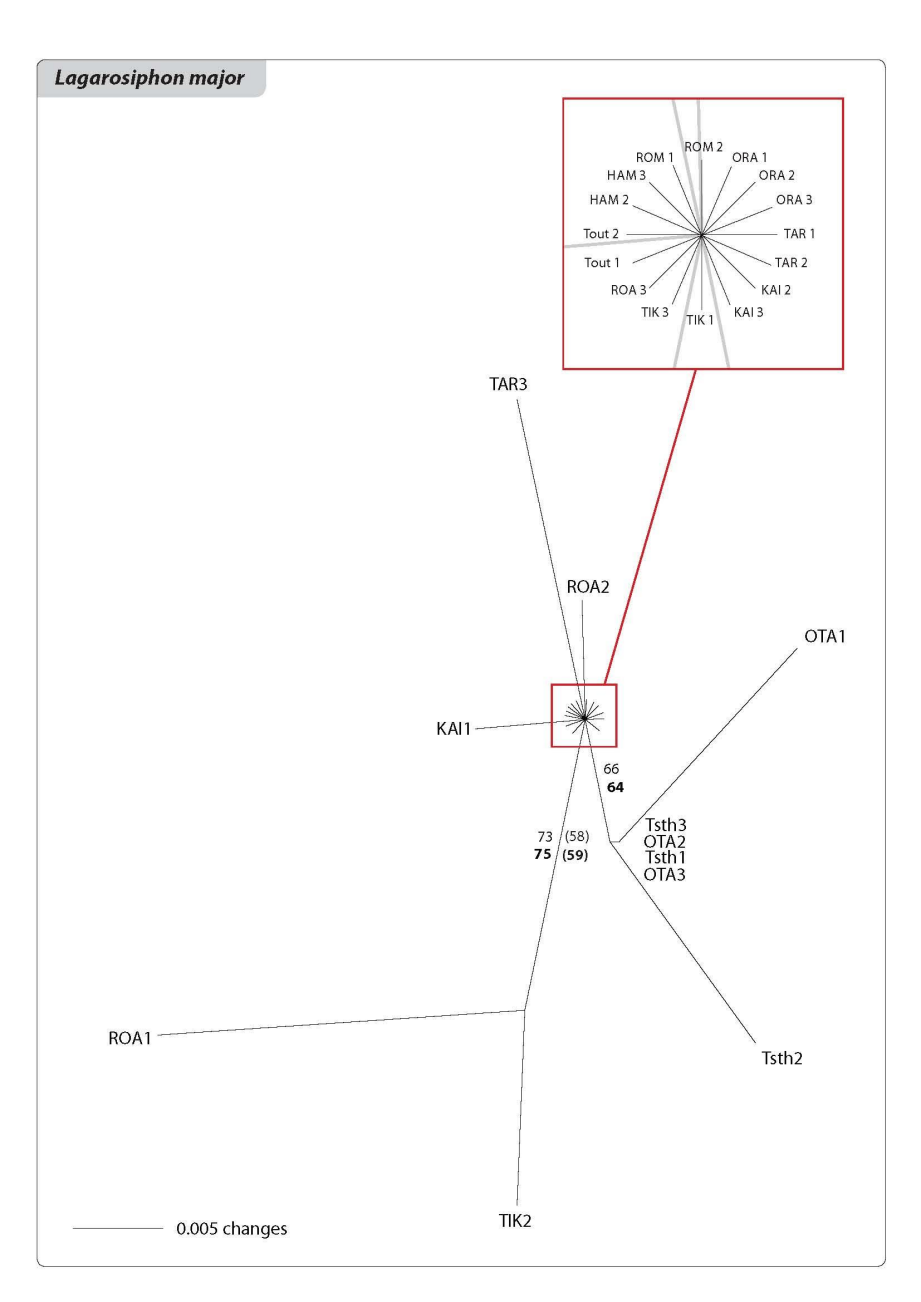
**Neighbour-Joining phylogram of *Lagarosiphon major***. Samples are labeled with population abbreviation (see Abbreviations) and sample number (1-3). Numbers are jackknife and bootstrap (in brackets) support values based on Nei and Li genetic distance (bold) and average pairwise difference.

**Figure 5:**
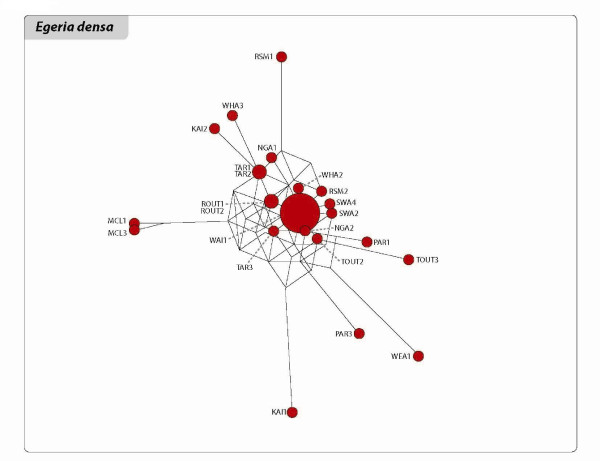
**Reduced median network of *Egeria densa***. The size of terminal nodes is proportional to genotype frequencies. Samples are labeled with population abbreviation (see Abbreviations) and sample number (1-3). The genotypes represented by the big terminal node are the same as those indicated in the small frame of Figure 6.

**Figure 6:**
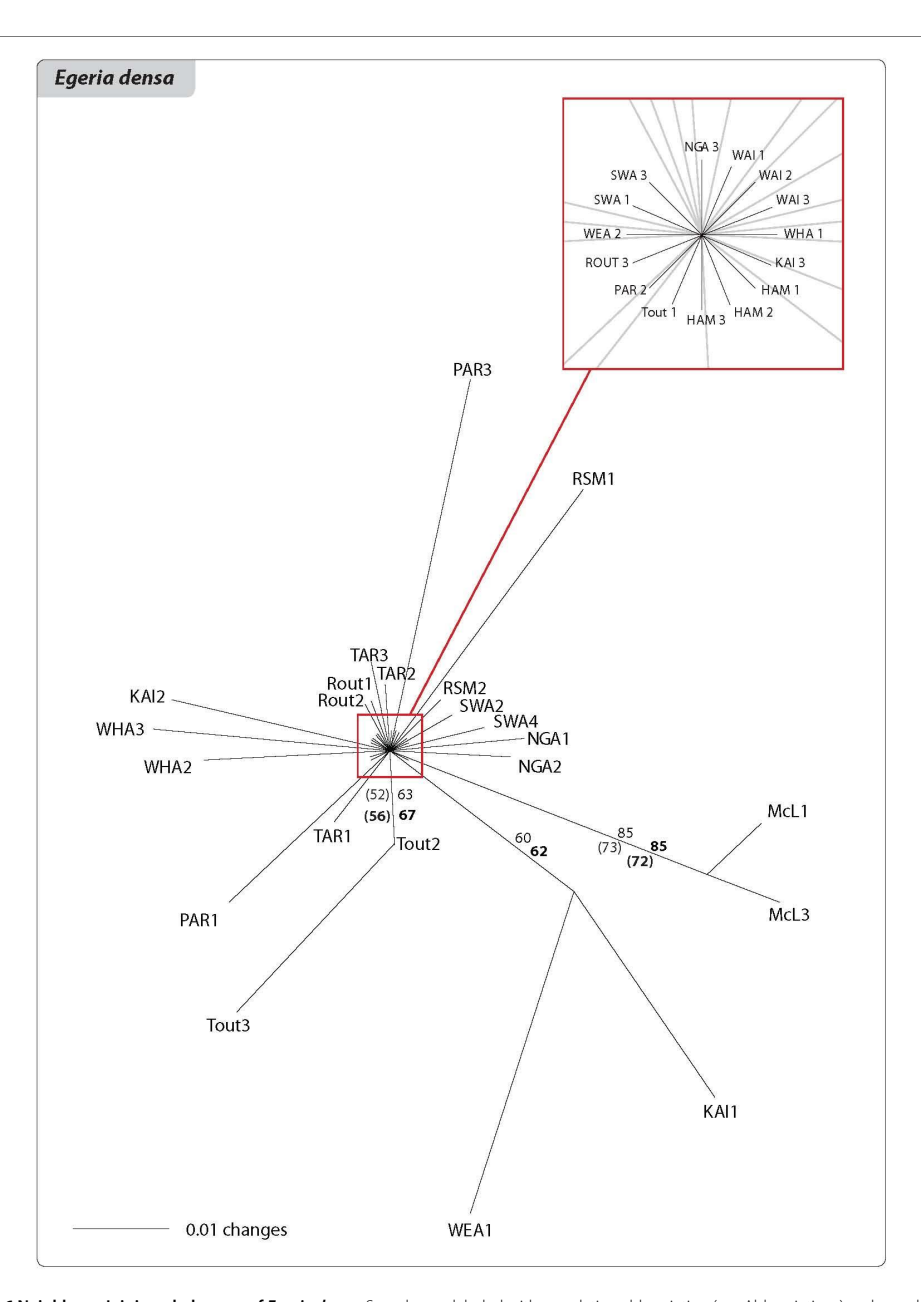
**Neighbour-Joining phylogram of *Egeria densa***. Samples are labeled with population abbreviation (see Abbreviations) and sample number (1-3). Numbers are jackknife and bootstrap (in brackets) support values based on Nei and Li genetic distance (bold) and average pairwise difference.

**Figure 7:**
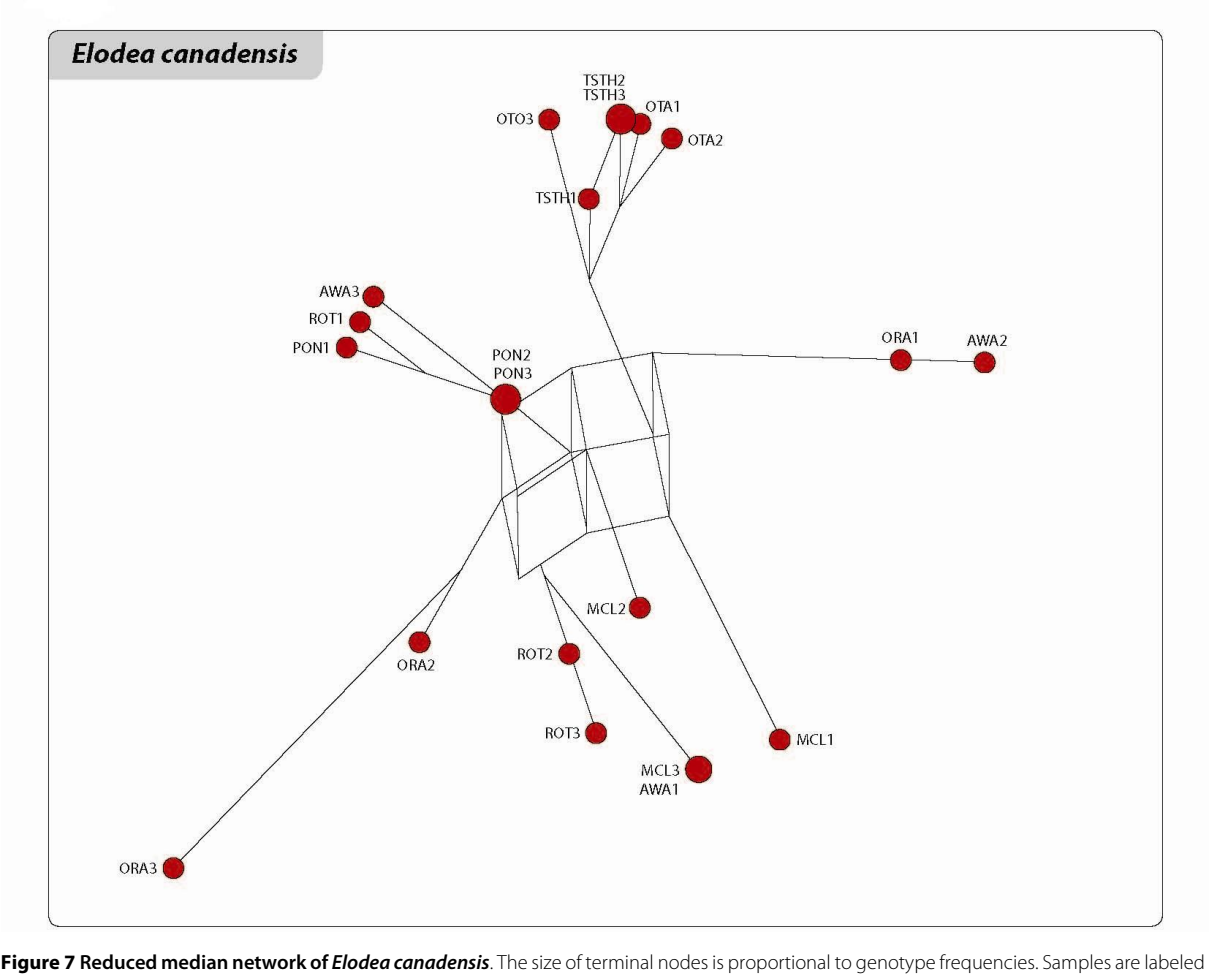
**Reduced median network of *Elodea canadensis***. The size of terminal nodes is proportional to genotype frequencies. Samples are labeled with population abbreviation (see Abbreviations) and sample number (1-3).

**Figure 8:**
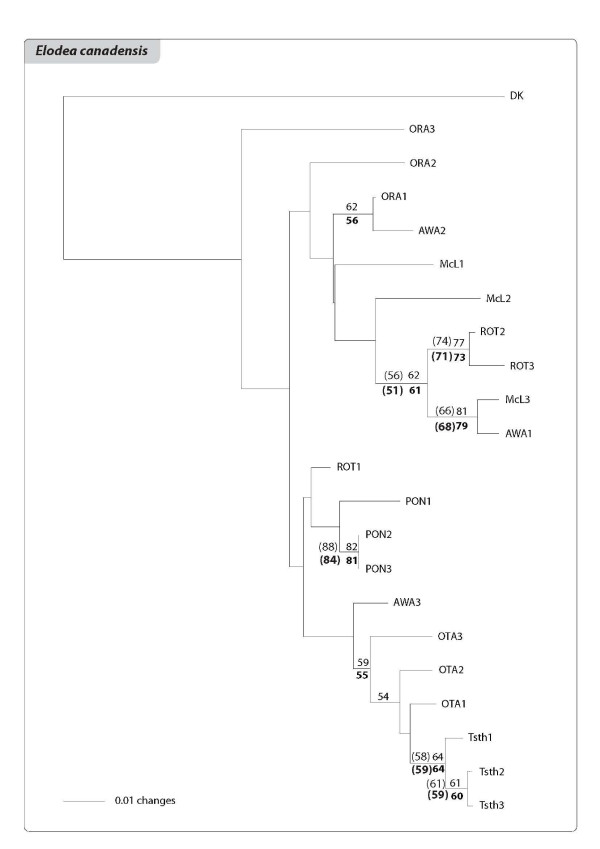
**Neighbour Joining phylogram of *Elodea canadensis***. One specimen of *E. canadensis *from Denmark (label abbreviation: **DK**) was used as an outgroup. NZ samples are labeled with population abbreviation (see Abbreviations) and sample number (1-3). Numbers are jackknife and bootstrap (in brackets) support values based on Nei and Li genetic distance (bold) and average pairwise difference.

The relationships among the clones of the three species are shown in the networks and Neighbour Joining (NJ) trees of Figures 3, 4, 5, 6, 7 and 8. The size of the terminal nodes in the network are proportional to the frequency of the genotypes and comparable in the three species. Although the samples of *E. canadensis *and *E. densa *showed similar levels of polymorphism (respectively 21.6% and 22.8% of the number of DNA fragments analysed) and higher than those of *L. major *(9.8%), the genetic pattern was different in the three species. In *L. major *the samples from the geographically close populations of Otamangakau and Taupo South appeared as a monophyletic group differentiated from a dominant genotype spread in every location (Figure 3). The interrelationships of Otamangakau and Taupo South clones, involving all samples from these populations, were supported by jack-knife values in the NJ tree (Figure 4). The other supported relationship in the NJ tree between two apparently very different and geographically distant clones collected at Rotoaira and Tikitapu lakes was also detected by the network, which introduced two common ancestral genotypes to explain their genetic affinities. The network of *E. densa *(Figure 5) showed a complex pattern of relationships and introduced a number of ancestral genotypes to connect the most different samples to the most spread clone, indicating a higher extent of differentiation than in *L. major*, as also evident by the larger number of polymorphic fragments. Apart from two clones collected at McLaren lake, whose relationship was confirmed also by jack-knife and bootstrap support in the NJ tree (Figure 6), *E. densa *populations did not appear to be genetically distinct. The network of *E. canadensis *showed a better defined structure than in *E. densa *and a number of monophyletic relationships among clones, including in many cases pairs of samples from the same populations or groups of genotypes from geographically close locations (Figure 7). As in *L. major*, the populations of Otamangakau and Taupo South appeared as a monophyletic group evolved from an ancestral genotype. The NJ tree provided jack-knife and/or bootstrap support for some of the relationships and showed a *continuum *of genetic differences among the samples and the populations of Otamangakau, Taupo South and Pongakawa (Figure 8).

**Figure 9:**
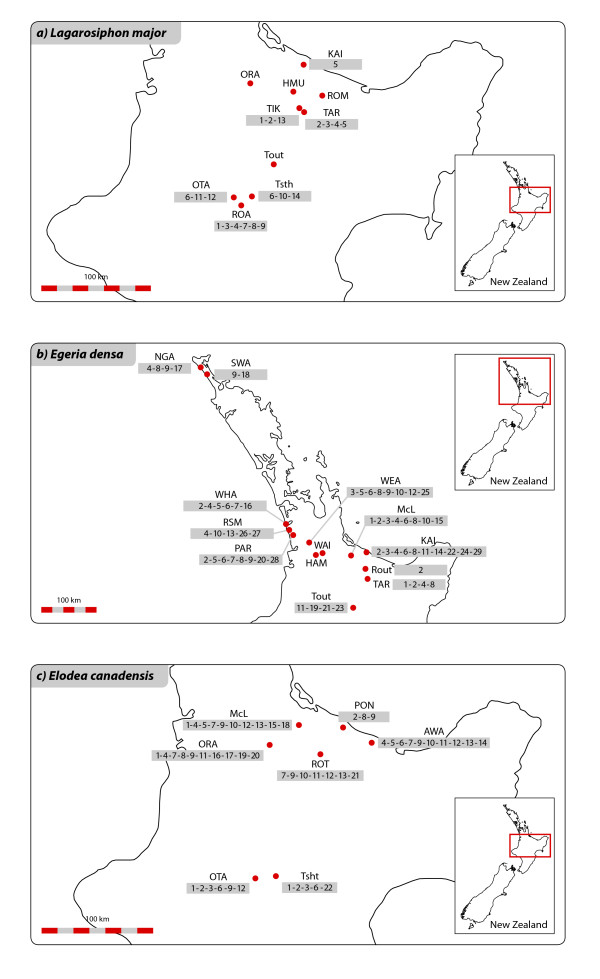
**Sampling locations for 9a) *Lagarosiphon major*, 9b) *Egeria densa*, 9c) *Elodea canadensis***. Locations abbreviations are listed in the Abbreviations section. The numbers in gray under the locality name indicate polymorphic DNA fragments present in the samples of that population. The same number in different populations refers to the same polymorphic fragment and is an indication of dispersal events between such populations.

Considering the low level of polymorphism observed in the three species, we assumed that individuals sharing the same polymorphic DNA fragments were either once integrated in the same clone or were closely related. We studied the distribution of polymorphic fragments throughout each dataset in order to track possible dispersal paths (Figure 9). This analysis showed many instances of polymorphic fragments shared between populations. It was not, however, possible to determine in which direction diaspores have been transported. In addition, samples with different combinations of polymorphic fragments were present in the same populations, indicating possible multiple introductions in lakes/rivers from different sources. In *L. major *(Figure 9a), four dispersal events could be reconstructed between Rotoaira and Tikitapu, Rotoaira and Tarawera, Tikitapu and Tarawera, and Tarawera and Kaituna. One polymorphic fragment was also shared by Otamangakau and Taupo South, indicating dispersal opportunities also between these two populations. The remaining polymorphic fragments were found in single samples only.

A larger number of polymorphic fragments was present in *E. canadensis *and *E. densa *and the interpretation of their patterns was less straightforward than in *L. major*. In *E. canadensis *(Figure 9c) the populations of McLaren, Oraka, Rotorua, Awakaponga and Pongakawa showed more frequent exchanges between each other than with the more distant, upstream populations of Otamangakau and Taupo South, which, likewise, were genetically more similar to each other than to the other populations. McLaren and Awakaponga had the highest number of polymorphic fragments shared with the other populations and appear to be important nodes for the dispersal and/or recruitment of this species. McLaren was found to be an important dispersal node also for *E. densa *(Figure 9b), and Parkinsons Lake for *E. densa *populations located in the NW part of the island. The Ngakeketa population from Northland shared polymorphic fragments with the neighbouring population of Swan and with Parkinsons Lake, but also with McLaren-connected populations, revealing long-distance dispersal. Long-distance dispersal appeared, however, less frequent than dispersal between neighbouring populations.

**Figure 10:**
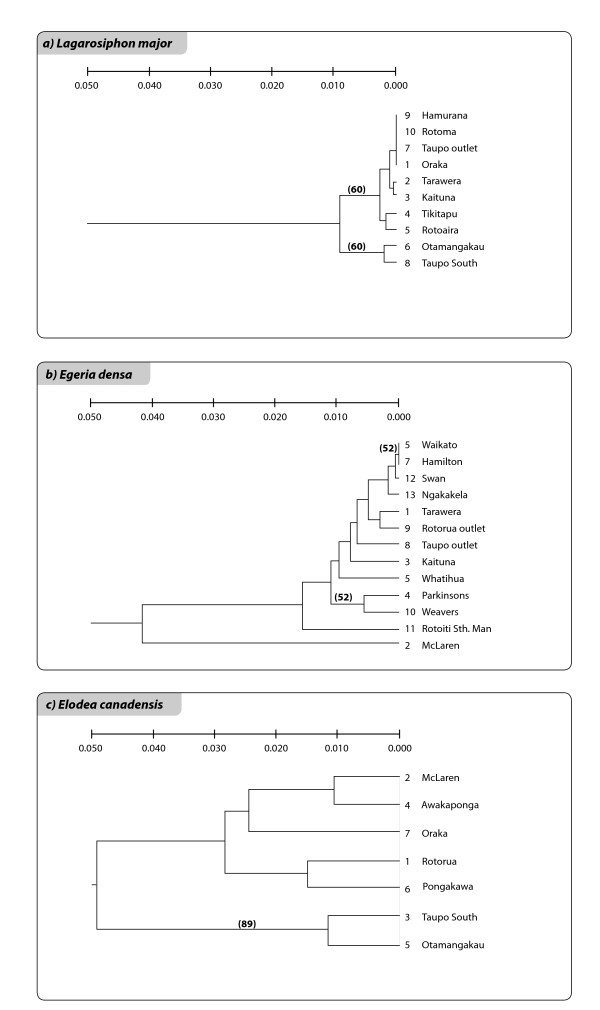
**UPGMA trees of 10a) *Lagarosiphon major*, 10b) *Egeria densa*, 10c) *Elodea canadensis *populations in NZ**. The highest bootstrap support value is indicated in each tree. The scale refers to Nei's unbiased minimum genetic distances. Terminal node numbers refer to the population numeration used for the analysis.

The UPGMA trees (Figure 10) show the genetic similarities between the populations of the three species. The lack of bootstrap support means that it cannot be excluded that the relationships shown are merely due to random "noise". However the topology of the trees did not change whatever the order of the samples in the data matrix, an indication that it is at least not entirely due to chance, but is a result of a history of several dispersal events and of genetic differentiation. In agreement with the higher Fst and Nei's unbiased minimum genetic distances calculated in *E. canadensis*, maximum genetic distances between *E. canadensis *populations were five times greater than between *L. major *populations and more than twice that between *E. densa *populations (excluding McLaren population), indicating a major extent of genetic structure in *E. canadensis *populations. The trees also supported the distinction between the upstream Otamangakau and Taupo South populations and the downstream populations both in *E.canadensis *and in *L. major*.

## Discussion

### AFLPs

The DNA quality, the reproducibility of AFLP chromatograms, the different patterns observed in *E. canadensis *compared to *E. densa *and *L. major *and the similar geographical separation of Otamangakau and Taupo South populations both in *E. canadensis *and in *L. major*, indicate that AFLPs are appropriate for the study of genetic variation in these clonal species. If the observed genetic patterns were due to artefacts of the AFLP technique, similar results would have been expected in the three species, considering the similarly low levels of genetic variation.

### Number of introductions

Low levels of genetic diversity were found in all three species, which suggest one introduction or multiple introductions of similar genotypes for each of the three species in the northern part of the North Island, NZ. High levels of genetic diversity in invasive species may be the result of multiple introductions [1,17]. However it is not possible to exclude multiple introductions even in cases with low genetic diversity, as the introduced genotypes might be highly similar to begin with. The genetic variation pattern in the native range is an important reference point of the natural variability within an invasive species and can support the hypotheses of single vs. multiple introductions. The situation in invasive species can, however, be more complicated than this, as they may be derived from areas in the introduced rather than the native range, which can be expected to give rise to different patterns. Such a high level of genetic similarity as that observed in this study was unexpected and is an important finding for further genetic research in these and other invasive species. A geographically wider set of samples, including many native and introduced populations, would need to be analysed to conclusively address the number of introductions in NZ. In the case of *L. major*, genetic variation within and between populations in the native range in South Africa was studied by Triest [18] using isozymes. Most of the populations were unisexual and monoclonal. Low variation levels were recorded also between populations, indicating predominant vegetative propagation. Evidence of sexual reproduction was provided by the presence of one heterozygotic clone in an otherwise homozygotic, bisexual population. The polymorphism between this clone and the most spread one was 9.5%, which, interestingly, is comparable with the level of polymorphism found in our study (9.8%). However, in the NZ populations this level of diversity was based on the whole sample set. The two most different genotypes had a polymorphism of 5.6%, and it decreased to 3.5% and 2.1% if differences between these clones and the most widespread one were considered. Our study shows lower levels of polymorphism than between the two different genotypes in the native range; however some more samples from South Africa should be analysed to estimate the variability in the native range. In addition, the different markers applied in the two studies (isozyme vs. AFLPs) are not totally comparable because the amount and the parts of the genome analysed in the two studies are very different and AFLPs are designed to detect higher levels of intraspecific polymorphism than isozymes.

Genetic diversity was studied in introduced populations of *E. densa *in Oregon (USA) and Chile using RAPDs by Carter and Sytsma [19]. Very little variation was found in these populations, and surprisingly the same genotypes were found in Oregon and Chile, suggesting low genetic diversity in the native source populations and/or similar introduction histories in the new ranges. Clones were genetically very similar to each other and the very short genetic distances between strains, as indicated by the UPGMA tree suggest a situation siMilar to that in the present study even though the molecular techniques used are different. The study of Carter and Systma [19] shows that multiple introductions of *E. densa *cannot be ruled out in the North Island, NZ. A stepping-stone model of colonization could, however, explain the similar genetic patterns in Oregon and Chile and hide higher levels of genetic diversity in the native range. In a study using allozymes, Kadono et al. [20] found genetically uniform populations of *E. densa *throughout Japan, but as in the case of isozymes, the technique yields a limited resolution compared to RAPDs and AFLPs.

To the best of our knowledge this is the first study of the genetic variation pattern of *E. canadensis *populations. In the congeneric invasive species *Elodea nuttallii *(Planchon) St John, both multiclonal [21] and monoclonal populations [20] were found in introduced ranges. In the Moder catchment (France), 2.7% AFLP polymorphism was detected in populations of *E. nuttallii *[22]. A single introduction was hypothesised and the low level of variation that did exist was attributed to somatic mutations. Interestingly, two different mutants were found in two of the ten investigated populations and, like in our study, the UPGMA tree showed geographical structure in the distribution of genetic variation. Multiple introductions in NZ from Tasmania were discussed by Thomson [15] for *E. canadensis*. If this is correct, genetic diversity had to be very limited in the source populations, suggesting a stepping-stone model of invasion in NZ. We do not know the genetic pattern in Tasmania and how many source populations were involved. We can only move the discussion about the number of introductions from NZ to Tasmania. The differences with the Danish genotype suggest that higher levels of DNA variation are present within the species, but *E. canadensis *is invasive also in Europe and different evolutionary patterns could explain the divergence of allopatric gene pools.

Not knowing the genetic variation of these species in their native range, we cannot draw conclusions about the number of introductions to NZ (or Tasmania). Two possible scenarios emerge, however, from this study: multiple introductions of genetically similar genotypes, or one single founding event (consisting either of one genotype or a set of genetically similar genotypes) and evolution *in situ *by somatic mutations. The first scenario would entail either low genetic diversity in the native range or a stepping-stone model of colonization from other areas of the introduced range, as appears to be the case for E*. canadensis*. The second scenario, involving evolution of genetic diversity in NZ by somatic mutations is consistent with our results. A third model, which parsimoniously combines the previous two, limiting both the number of dispersal events and the number of mutations to have occurred in NZ, is that of ancestral polymorphisms retained in the three species [23].

### Sources of genetic variation

Several results of this study indicate that evolutionary processes occurred after these species were introduced in the northern part of NZ's North Island. The measures of population differentiation show that the time elapsed since introduction is reflected in the genetic structure of the populations. *Elodea canadensis *was the first of the three species to be introduced in 1868 and its populations have higher Fst values and Nei's genetic distances than the later-established populations of *E. densa *and *L. major*. Also, the pattern of *E. canadensis *differs from that of the other species in showing more monophyletic groups, a *continuum *of genetic differences between individuals and populations, and differentiation between the geographically close populations of Taupo South and Otamangakau from the downstream locations. Strikingly the populations of Taupo South and Otamangakau were found to be a monophyletic group also in *L. major*, which was introduced in NZ as recently as in 1950. This can be explained either by recent "co-dispersal" of the two species (after 1950), or by independent dispersal histories shaped by the same geographic/physical constrains. In both cases multiple independent introductions appear very unlikely, as those would have blurred any common pattern. Considering that only one sex is present, or dominant, in each species, the absence of seeds and evident outcrossing events (recorded in the spectrum of pairwise genetic distances), somatic mutations seem to be a plausible source of genetic diversity in these clonal species.

The spectrum of pairwise genetic distances has previously been utilized to study genetic diversity in populations of clonal outcrossing plant species, as it may be helpful in distinguishing between genetic variation produced by somatic mutations and genetic variation due to outcrossing. The spectrum of clonal outcrossing populations of the marine submerged *Posidonia oceanica *(L.) Del. [24] shows a bimodal distribution, with a large peak at zero pairwise distance (α peak) indicating clonal reproduction, followed by decreasing frequencies in pairwise distances, due to somatic mutations. The second peak of the spectrum (β peak) is the mode of an almost normal distribution of frequencies in pairwise distances, which has been attributed to genetic diversity produced by outcrossing events. The same spectrum was obtained in outcrossing populations of the clonal tree *Populus tremuloides *Michaux [25]. Compared to these studies, the spectra of *L. major *and *E. densa *populations show the same pattern that has been considered indicative of clonal reproduction and somatic mutations. Contrastingly, the spectrum of *E. canadensis *populations does not fit with this model. The normal distribution of pairwise differences points to outcrossing genotypes, whereas the limited range of polymorphism, which is comparable to that of *E. densa*, points to somatic mutations. The comparison with a genotype from Europe shows higher numbers of polymorphic fragments with all NZ genotypes. In addition if the spectrum was the result of outcrossing events between the NZ genotypes, outcrossing would be more frequent than clonal propagation, as the frequency of "zero" pairwise differences is very low, and seeds would be common in the populations. Seeds have, however, never been observed in *E. canadensis *in the northern part of NZ's Northern Island [14]. However, the lack of β peak is not a conclusive evidence of absence of outcrossing. Sexual reproduction between clonal stands with the same or highly similar genotypes is not supposed to produce a β peak. Pairwise difference frequencies are also affected by somatic mutation rates as well as by population sizes at the time of introduction and at present, and by genetic diversity at the time of introduction. Several factors might interact in *E. canadensis*'s spectrum, such as a pool of genetically similar/closely related founders, possible mutation-drift disequilibria and a longer introduction time.

Genetic diversity levels are different in the three species, in relation to the initial gene pools, possible ancestral polymorphisms, different somatic mutation rates and establishment times. *Egeria densa*, which in the North Island of NZ has the typical spectrum of a clonally reproducing species that has accumulated somatic mutations, has more genetic diversity than the other macrophytes. This makes *E. densa *more adaptable. In invasive populations of the clonal aquatic species *Hydrilla verticillata *(L. f.) Royle, Albrecht et al. [26] demonstrated that herbicide resistance evolved by somatic mutations.

### Dispersal

In the absence of evident sexual reproduction, the distribution of genetic diversity in the populations documented in this study is found to be due to the dispersal of vegetative propagules, moderated by the presence of possible geographic/ecological barriers. The analysis of the geographic distribution of DNA polymorphic fragments showed more frequent dispersal events between neighbouring populations, even though long-distance dispersal was also common. Multiple introductions in lakes and rivers appeared also to be a recurring phenomenon, as well as down- and up-stream dispersal. The co-occurrence of more invasive species in the same localities and similarly complex dispersal routes in all three species suggest that human dispersal had a major role in the distribution of the genetic diversity. Johnstone et al. [27] ruled out bird-mediated dispersal because the distribution patterns of these species in NZ were not random in nature, but linked to fishing and boating activities. Considering that the populations sampled in this study were from a wide range of different habitats [28], dispersal opportunities combined with an empty niche [29] rather than suitable environmental conditions, explain the distribution of these species in the northern part of NZ's North Island, as also found by de Winton et al. [12]. As discussed by Howard-Williams [30], NZ has no native canopy-forming submerged aquatic plants. Some kind of barrier seems, however, to limit recruitment and dispersal from Otamangakau and Taupo South from/to downstream populations in *E. canadensis *and *L. major*. Surprisingly, in *L. major*, these populations are isolated also from Lake Rotoaira, which is geographically close and connected by waterways to Otamangakau. A wider set of samples and populations covering also the southern part of the North Island and the South Island, combined with historic records of first introduction date in each location would help better understand the relationships between populations and reconstruct the introduction history of these species in NZ with higher resolution. It would also greatly enhance interpretation of nodes of dispersal vs. recruitment, providing a valuable tool for the local management of these aquatic weeds.

## Conclusions

Very low levels of genetic diversity were found in three invasive aquatic species in the northern part of North Island, NZ. Even though it is not possible to quantify how much of the genetic variation can be attributed to each founding event, this study provides some evidence that evolutionary changes have occurred in the introduced range, even though sexual reproduction is not frequent or does not occur at all. Post-introduction evolution is supported by population genetic structure and similar phylogeographic patterns in *E. canadensis *and *L. major*. Considering the lack of seeds and the random dispersal of vegetative propagules, which appears to be human-mediated, somatic mutations are supposed to be a potential source of genetic diversity in these clonal species. However, this study does not provide direct evidence of such evolutionary events. Genetic variation should be analysed in the native populations to conclusively shed light on the evolution of these species in the North Island of NZ.

## Methods

### Sampling

We sampled populations of the three species in a number of locations in the northern part of the North Island, NZ (Figure 9). This sampling area was chosen based on floristic and ecological records which indicate that all three species were established in this area at early stages of their introductions and from here their rapid spread was followed throughout the North Island [12]. We focused on this region to test to what extent, in the absence of sampling across continents, a set of molecular data collected from a restricted geographic area can be informative about introduction history, and specifically the number of introductions. We used a sample of *E. canadensis *collected in Denmark as a reference to evaluate the genetic similarities of the NZ populations against a different genotype.

At each location three samples were collected from different macrophyte beds randomly chosen at an average distance of about three metres. As clonal propagation is the main (or exclusive) form of reproduction in these species in North Island, we considered a geographical extension of the sampling area more informative than an increased number of samples collected at each location. The term population is used in this study in a broader sense, which includes perennial clonal stands that are not necessarily in a gene flow relationship. From each specimen we sampled the top shoot and stored it in a sealed bag with silica gel. Samples from the same locations were considered representative of one population. In total 21 specimens of *E. canadensis *, representing 7 populations, 42 specimens of *E. densa *(14 populations) and 30 specimens of *L. major *(10 populations) were AFLP fingerprinted.

### DNA Extraction

DNA was extracted with the E.Z.N.A. plant kit from the apical parts of shoots. DNA quality was checked on a 0.8% agarose gel, run for 1 h at 120V. DNA consisted of a sharp band of the size of about 20.000 bp compared to the Lambda DNA, Hind III marker 2 (MBI Fermentas) and no signs of DNA digestion were visible.

DNA concentration was measured with the Nano Drop Spectrophotometer ND-1000 (Saveen Werner) at 280 nm wavelength and was between 50 and 200 ng/μl in all samples, eluted with 50 μl E.Z.N.A. elution buffer.

### DNA restriction and ligation

20 ng DNA were used for DNA restriction with 1.25 units each of EcoRI and MseI enzymes and ligation to 2.5 pmol EcoRI and 25 pmol MseI nucleotide adapters with 335 NEB cohesive end ligation units. Restriction and ligation were carried out simultaneously in a PCR (Peltier Thermal Cycler PTC-200 - MJ Research). The program was 37 ˚C for 4 h followed by 0.1 ˚C decrease in 2 h and 10 min at 70 ˚C. Restricted and ligated DNA was diluted 4x prior to pre-amplification.

### Pre-amplification

2 μl of template DNA were added to 18 μl mastermix consisting of 10 μl 2x Mastermix (VWR Ampliqon), 6 picog EcoRI primer, 6 picog MseI primer and sterile water to reach the final volume of 20 μl. EcoRI primer and MseI primer consisted of one-nucleotide selective base. The PTC-200 PCR was programmed for 20 cycles, each with a 30 s DNA denaturation at 94 ˚C, 1 min primer annealing at 56˚C, and 1 min extension at 72 ˚C. Preamplified DNA was diluted 4x prior to selective amplification.

### Selective amplification

2 μl of template DNA were added to 18 μl mastermix consisting of 10 μl 2x Mastermix (VWR Ampliqon), 1 picog EcoRI primer, 6 picog MseI primer and sterile water to reach the final volume of 20 μl. EcoRI primer was Cy (fluorochrome) labelled. The PCR program was 94˚C for 30 s, 65 ˚C for 30 s decreased by 0.7 ˚C/cycle for the subsequent 12 cycles, 72 ˚C for 1 min, followed by 23 cycles at 94 ˚C for 30 s, 56 ˚C for 30 s and 72 ˚C for 1 min. Several primer combinations were tested and many did not show polymorphism. Three primer combinations were selected for *E. canadensis *(E-ACTcy+M-CTC, E-ATGcy+M-ACG, E-CGTcy+M-AGC) and *E. densa *(E-ACTcy+M-CTC, E-ATGcy+M-ACG, E-CAGcy+M-TAC) and four primer combinations for *L. major *(E-ACTcy+M-CTC, E-ATGcy+M-ACG, E-CGTcy+M-AGC , E-CAGcy-M-GCA) based on the presence of polymorphic DNA fragments and the scoring possibilities.

### Electrophoresis

was run on a 7% acrylamide gel (Reprogel - Long Read) with 50-500 bp external sizers (GE Healthcare), in an ALF Express II DNA Analysis System (Amersham Pharmacia Biotech) at 1500 V, 55˚C for 400 min. 5 μl amplified DNA were added to 3 μl loading dye (GE Healthcare) and DNA was denatured in the PTC-200 PCR for 5 min at 94˚C prior to loading on the gel.

### AFLPs scoring

DNA fragments between 50 and 500 bp were scored for presence or absence with the program ALFwin Fragment Analyser Software package (Amersham Pharmacia Biotech). Only peaks clearly above the detection limit (set with a peak shape of 10 and a minimum height of 1%), well amplified and resolved in all samples were scored for polymorphism. The AFLP chromatograms were virtually identical within each species, with the exception of some samples in which a few peaks were absent (or, in a few cases, present) compared to the rest of the sample set. DNA was extracted, digested, ligated and amplified twice from these samples, to make sure that missing DNA fragments were due to polymorphism and not to DNA fragmentation. The complete reproducibility of AFLP chromatograms excluded possible ligation [31] and PCR [32] artefactual DNA fragments. A few samples of each species produced interrupted chromatograms and were excluded from the dataset. In total 102 DNA fragments were scored in 22 samples of *E. canadensis *, 127 DNA fragments were scored in 38 samples of *E. densa *and 142 DNA fragments were scored in 27 samples of *L. major*.

The scored polymorphic loci clearly showed either high peaks or no peaks. Low intensity peaks occurred in a very limited number of polymorphic loci and were scored as question marks. They corresponded to 1.5% of the peaks scored in *E. canadensis*, 1% of the peaks scored in *E. densa *and 0.7% of the peaks scored in *L. major*. These levels of ambiguity are comparable to or lower than those quantified by Bonin et al. [33] as typical systematic genotyping errors associated with the scoring of AFLP data. In *E. canadensis *nine polymorphic fragments, out of 22, had a frequency over 0.20 and one fragment had a frequency of 0.44. Polymorphic fragments were in most cases missing or present in all samples from the same population. In *E. densa *and *L. major *datasets only 3 and 1 DNA fragments respectively had a frequency over 0.20 and it was not possible to recognize any "population pattern" by scoring the gels.

### Data analysis

The resulting binary matrix was analysed for each species with the program Arlequin ver. 3.11 [34] to estimate the extent of genetic variation contained within and among populations and the extent of genetic differentiation of the populations. Question marks in the matrix were treated as missing data. AMOVA and population comparison test were based on pairwise differences and were tested respectively with 1000 and 100 permutations [35]. The exact test of population differentiation, based on haplotypic frequencies, was calculated with a Markov chain of 100,000 steps and 10,000 dememorization steps [35]. The same analyses were carried out between all NZ samples of *E. canadensis*, considered as one population, and the Danish genotype. Population differentiation was also evaluated based on Nei's [36] unbiased minimum genetic distances, which are limitedly affected by differences in sample size [37] and unbiased by small sample sizes [36]. The genetic distances were calculated with the program TFPGA ver. 1.3, 2000 (Tools For Population Genetic Analyses; [38]). Question marks in the matrix were treated as missing data.

Genetic relationships among clones were calculated with PAUP ver. 4.0b10 (Phylogenetic Analysis Using Parsimony; [39]) and with Network ver. 4.5.1.6 Phylogenetic Network Constructions (Copyright 2004-2010 Fluxus Technologies Ltd.). PAUP NJ analysis were carried out based on both mean character difference in number of polymorphic fragments and genetic distances [40]. Question marks were fitted by the program, either as present or absent fragments, in order to minimize tree length (lowest mean character difference or shortest genetic distance). Jack knife support was obtained with 37% character deletion [41] with 1000 replicates, "emulate jac-resampling" and random number seed. Bootstrap was set with 100 character resampling. The Danish genotype of *E. canadensis *was used as the outgroup for the NZ samples. For *E.densa *and *L. major *the NJ trees were calculated as unrooted phylograms.

The network of all maximum parsimonious trees was constructed with the reduced median algorithm [42]. This algorithm, originally developed for binary matrices obtained by haploid sequence data, could be used for AFLP data because all characters could be entered with the same weight which is possible when the number of samples does not exceed 50. Question marks were processed by the program either as present or absent fragments to reach the shortest tree, estimated as lowest number of mutations. Using a parsimony criterion, the program introduces hypothetic missing haplotypes to the network, represented as median vectors (nodes).

We used the program TFPGA also to calculate UPGMA trees of the populations. Nei's genetic distances (unbiased minimum genetic distances) are based on pairwise common DNA polymorphic fragments and similarities between populations can be assumed to reflect a possible common vegetative origin and/or dispersal opportunities between populations. Statistical support was assessed with bootstrap analysis based on 1000 permutations and by changing the order of the samples several times in the data matrix, in order to alleviate the taxon entry-order problems associated with UPGMA method, as pointed out by Backeljau et al. [43].

## Abbreviations

Localities abbreviations: **AWA**: Awakaponga; **HAM**: Hamilton; **HMU**: Hamurana; **KAI**: Kaituna; **McL**: McLaren; **NGA**: Ngakeketa; **ORA**: Oraka; **OTA**: Otamangakau; **PAR**: Parkinsons; **PON**: Pongakawa; **ROA**: Rotoaira; **ROM**: Rotoma; **ROT**: Rotorua; **Rout**: Rotorua outlet; **RSM**: Rotoiti Sth. Man; **TAR**: Tarawera; **Tout**: Taupo outlet; **TIK**: Tikitapu.; **Tsth**: Taupo South; **SWA**: Swan; **WAI**: Waikato; **WEA**: Weavers; **WHA**: Whatihua.

## Authors' contributions

CL ran the AFLPs, analysed the data and wrote the manuscript. TR coordinated the study and prepared the figures. TR, BO and JSC collected samples in New Zealand. All authors contributed to the scientific discussion of the results, commented the draft manuscript, and read and approved the final manuscript.
